# Hypoxia‐related lncRNAs to build prognostic classifier and reveal the immune characteristics of EGFR wild type and low expression of PD‐L1 squamous and adenocarcinoma NSCLC

**DOI:** 10.1002/cam4.4126

**Published:** 2021-07-11

**Authors:** Fang Zhao, Min Wang, Jie Zhu

**Affiliations:** ^1^ Department of Intensive Care Unit The Peoples Hospital of Tongliang District Chongqing China; ^2^ Department of Respiratory and Geriatrics Chongqing Public Health Medical Center Chongqing China; ^3^ Department of Respiratory and Critical Care Medicine West China Hospital Sichuan University Chengdu China

**Keywords:** EGFR wild type, hypoxia, lncRNAs, non‐small cell lung cancer, PD‐L1, TCGA

## Abstract

**Background:**

Recently, the development and application of targeted therapies like tyrosine kinase inhibitors (TKIs) and immune checkpoint inhibitors (ICIs) have achieved remarkable survival benefits in non‐small cell lung cancer (NSCLC) treatment. However, epidermal growth factor receptor (EGFR) wild type and low expression of programmed death‐ligand 1 (PD‐L1) NSCLC remain unmanageable. Few treatments for these patients exist, and more side effects with combination therapies have been observed. We intended to generate a hypoxia‐related lncRNAs (hypolncRNAs) classifier that could successfully identify the high‐risk patients and reveal its underlying molecular immunology characteristics.

**Methods:**

By identifying the bottom 25% PD‐L1 expression level as low expression of PD‐L1 and removing EGFR mutant samples, a total of 222 lung adenocarcinoma (LUAD) and lung squamous carcinoma (LUSC) samples and 93 adjacent non‐tumor samples were finally extracted from The Cancer Genome Atlas (TCGA). A 0 or 1 matrix was constructed by cyclically pairing hypoxia‐related long non‐coding RNAs (hypolncRNAs) and divided into the train set and test set. The univariate Cox regression analysis determined the prognostic hypolncRNAs pairs. Then, the prognostic classifier contained nine hypolncRNAs pairs which were generated by Lasso regression and multivariate Cox analysis. It successfully stratified EGFR wild type and low expression of PD‐L1 squamous and adenocarcinoma NSCLC (double‐negative LUAD and LUSC) patients into the high‐ and low‐risk groups, whose accuracy was proved by the time‐dependent receiver operating characteristic (ROC) curve. Furthermore, diverse acknowledged immunology methods include XCELL, TIMER, QUANTISEQ, MCPcounter, EPIC, CIBERSORT‐ABS, CIBERSORT, and the single‐sample gene set enrichment analysis (ssGSEA) revealed its underlying antitumor immunosuppressive status in the high‐risk patients.

**Conclusions:**

It is noteworthy that hypolncRNAs are associated with the survival of double‐negative LUAD and LUSC patients, for which the possible mechanism is inhibiting the antitumor immune process.

## Graphical abstract

We conducted a comprehensive analysis of hypoxia‐related lncRNAs and revealed potential inflammatory mechanism in epidermal growth factor receptor wild type and low expression of programmed death‐ligand 1 squamous and adenocarcinoma non‐small cell lung cancer based on the Cancer Genome Atlas database.

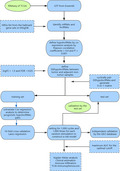

## 1 INTRODUCTION

Over the years, oncogenic molecular alterations such as epidermal growth factor receptor (EGFR) were widely recognized in non‐small cell lung cancer (NSCLC) and contributed to clinical treatment. Extensive somatic mutations in the EGFR gene were observed in Caucasian and East Asian lung adenocarcinoma (LUAD) patients (approximately 10% and 50%, respectively).^1^, ^2^ The EGFR tyrosine kinase inhibitors (TKIs) that target EGFR mutation have been proved to be one of the most effective treatment options. Advanced NSCLC patients with EGFR mutation have achieved noticeable survival improvement compared with platinum‐based chemotherapy.^3^, ^4^, ^5^ Meanwhile, immune checkpoint inhibitors (ICIs) targeting programmed death receptor‐1 (PD‐1) and programmed death receptor ligand‐1 (PD‐L1) also achieved advanced NSCLC. By binding to PD‐L1 expressed on the surface of tumor cells, PD‐1 mediates the T‐cell inactivation, leading to the immune escape of tumor cells.^6^ Pembrolizumab, a PD‐1 inhibitor, has shown a more effective response in tumor PD‐L1 overexpression of at least 50% of patients than first‐line platinum doublet chemotherapy.^7^

However, TKIs were less useful for EGFR wild‐type patients. And predominant patients with EGFR mutation developed resistance with TKIs in 12 months.^4^ The clinical efficacy of ICIs was also related to PD‐L1 expression. Patients with higher tumor and immune cell PD‐L1 expression got more treatment benefit from ICI atezolizumab.^8^, ^9^ When the expression of PD‐L1 was between 1% and 50%, ICIs showed less therapeutic benefit and more adverse events.^9^ This led to the use of TKIs and ICIs restricted to a small percentage of patients, leading to the majority of patients with EGFR wild type and low expression of PD‐L1, the double‐negative LUAD and lung squamous carcinoma (LUSC), remain in the treatment dilemma. Searching for more effective therapeutic markers is urgent.

Hypoxic microenvironments are formed when the demand of cancer cells exceeds intravascular oxygen supply, leading to hypoxia‐related gene (HRG) expression fluctuations.^10^ The hypoxic microenvironments are essential factors affecting cancer cell phenotype and behavior,^11^ affecting prognosis and treatment response. Hypoxia is reported to trigger tumor immunosuppression via inhibition of T‐cell proliferation and upregulation of co‐inhibitory receptors or recruitment of immunosuppressive cells.^12^, ^13^, ^14^, ^15^ Long non‐coding RNAs (lncRNAs) are a class of RNAs larger than 200 nucleotides and cannot encode proteins,^16^ which are also involved in tumor immunodeficiency. For example, lncRNA Lnc‐Tim3 intensifies CD8 T‐cell exhaustion in hepatocellular carcinoma.^17^ lncRNA SNHG1 regulates Treg cell differentiation and leads to immune escape in breast cancer.^18^ lncRNA NKILA mediates T‐cell sensitivity to tumor cell death, playing an immunosuppressive role in tumor immunology.^16^

We intended to reveal immunologic disturbances of the double‐negative LUAD and LUSC and identify new treatment markers in view of the low responsiveness of double‐negative LUAD and LUSC to therapy. In the present work, we proposed a hypoxia‐related lncRNAs (hypolncRNAs) prognostic classifier using public RNA sequencing data from The Cancer Genome Atlas (TCGA). The current immune analysis methods such as XCELL, TIMER, QUANTISEQ, MCPcounter, EPIC, CIBERSORT‐ABS, CIBERSORT, and the single‐sample gene set enrichment analysis (ssGSEA) analysis were used to reveal tumor‐infiltrating immune cells disturbances between subgroups. Considering the two‐biomarker combination strategy has higher accuracy in prognosis prediction,^19^ we used a new algorithm, pairing two lncRNAs as an integrated biomarker, in which the specific expression levels were not required, to construct the prognostic classifier.

## 2 METHODS

### 2.1 Preparation of data and differentially expressed analysis

The present study included LUAD and LUSC patients from a public dataset, transcriptome profiling (RNAseq) data, mutation data, and clinical data were retrieved from TCGA (https://tcga‐data.nci.nih.gov/tcga/). The probe data were transformed into fragments per kilobase million (FPKM). Gene expression profiles were summarized to provide gene‐level information after the microarray probes (raw data profiles) were mapped to gene symbols depending on their chips and platform, which was annotated by gene transfer format files downloaded from Ensembl (http://asia.ensembl.org). The HRGs list was downloaded from the hallmark gene sets in the Molecular Signature Database (MSigDB) (https://www.gsea‐msigdb.org/gsea/msigdb/). We defined the lncRNAs that satisfied the Pearson correlation coefficients >0.4 and *p* < 0.001 between them and HRGs as hypolncRNAs. The differentially expressed HRGs (DEHRGs) and hypolncRNAs (DEhypolncRNAs) between tumor and adjacent non‐tumor samples were screened out when both log fold change (FC) >1.5 and false discovery rate (FDR) <0.05 were satisfied.

### 2.2 Definition of double‐negative LUAD and LUSC and pairing DEhypolncRNAs

By excluding samples of EGFR mutations, the TCGA cohort comprised 904 EGFR wild‐type samples and 93 adjacent non‐tumor samples. PD‐L1 low expression was defined as the bottom 25% expression level. A total of 222 tumor samples were finally identified as EGFR wild‐type and low expression of PD‐L1 samples.

A 0 or 1 expression matrix was established by cyclically pairing all the DEhypolncRNAs. The value of the DEhypolncRNAs pair was equal to the value of lncRNA A plus lncRNA B. When the expression level of lncRNA A was higher than that of lncRNA B, the pair value was defined as 1; otherwise, it was defined as 0. We considered that a pair without a certain value fluctuation could not correctly predict the prognosis. A pair is considered valid when more than 20% of a single DEhypolncRNAs pair have a value of 0 or 1. A total of 17,534 valid DEirlncRNAs pairs were finally generated, randomly divided into the train set and test set. The prognostic classifier was generated from the train set and then verified by the test set.

### 2.3 Establishment of the prognostic classifier

We used the univariate Cox regression analysis to filtrate the prognostic DEhypolncRNAs pairs from the 0 or 1 expression matrix. The 10‐fold cross‐validation least absolute shrinkage and selection operator (Lasso) regression was set for a 1000‐cycle and every random stimulation for 1000 times, of which the multivariate Cox analysis was performed on DEhypolncRNAs pairs satisfying the frequency more than 100 times. Thus, a prognostic classifier was constructed with certain regression coefficients. The highest point of receiver operating characteristic (ROC) curve indicates the maximum area under the curve (AUC) was selected as the cutoff of the classifier values. Samples with a classifier index higher than the cutoff value were considered as high‐risk. The Kaplan–Meier analysis showed the survival differences between high‐ and low‐risk groups. The independent predictive ability of the prognostic classifier with clinical parameters including age, gender, and pathological stage were tested by the univariate Cox regression and the multivariate Cox regression analysis. Furthermore, the Kaplan–Meier analysis and ROC curves of the same classifier were validated under diverse clinical conditions. The R packages survival, glmnet, survivalROC, and survminer were utilized in this procedure.

### 2.4 Functional annotation and principal component analysis

Through Gene Ontology (GO) enrichment and Kyoto Encyclopedia of Genes and Genomes (KEGG) pathway analysis, we explored the potential biological functions of HRGs in tumorigenesis. Major biological attributes identified in GO and KEGG were determined and visualized by the R package clusterProfiler and GOplot. To determine whether the prognostic classifier accurately differentiated patients at different risks, principal component analysis (PCA) was performed respectively to expression profiles and different risk groups. The R package scatterplot3d accomplished three‐dimensional PCA plots.

### 2.5 Investigation of tumor‐infiltrating immune cells in microenvironment

Current acknowledged methods such as XCELL, TIMER, QUANTISEQ, MCPcounter, EPIC, CIBERSORT‐ABS, and CIBERSORT were united to reveal the immunologic characteristics between groups. Diverse immune‐infiltrating cells were estimated by Spearman correlation analysis and Wilcoxon signed‐rank test with classifier index and risk groups. The estimation files for the TCGA project to calculate the immune infiltration statues were downloaded from the TIMER website (http://timer.comp‐genomics.org). The R packages ggplot2, ggtext, scales, and limma were used in this procedure.

The ssGSEA is an expanded version of Gene Set Enrichment Analysis (GSEA), which classifies gene sets with common immune biological roles and physiological functions.^20^, ^21^ A total of 782 immune‐related genes were divided into 29 gene sets based on current information,^22^ representing specific immune cell populations and functions. The 29 gene sets were obtained: aDCs (activated dendritic cells), antigen‐presenting cell (APC) co‐inhibition, APC co‐stimulation B cells, CC chemokine receptor (CCR), Check‐point, CD8+ T cells, cytolytic activity, dendritic cells (DCs), human leukocyte antigen (HLA), interdigitating dendritic cells (iDCs), inflammation‐promoting, mast cells, macrophages, MHC class I, NK cells, neutrophils, para‐inflammation, plasmacytoid dendritic cells (pDCs), T‐cell co‐inhibition, T helper cells, T‐cell co‐stimulation, follicular helper T cells (Tfh), Th1 cells, tumor‐infiltrating lymphocytes (TIL), Th2 cells, Type I IFN response, regulatory T cells (Treg), and Type II IFN response.

### 2.6 Validation of the prognostic classifier in The Cancer Immunome Atlas

The Cancer Immunome Atlas (TCIA) is an online searchable database that enables researchers to develop and test hypotheses about the impact of cancer genomes on tumor microenvironment and immune characteristics, particularly with regards to ICIs treatment responses. The immunophenotypes of 20 solid cancers in TCGA were determined by the cellular characteristics of the immune infiltrates, suggesting a potential mechanism of tumor escape. Machine learning methods were used to identify tumor immunogenicity and generate an immunophenotypic scoring scheme. The immunophenoscore can be used as a good predictor of cytotoxic anti‐T‐lymphocyte‐antigen‐4 (anti‐CTLA‐4) and anti‐PD‐1 antibodies responses, which have been validated in two independent cohorts. A higher immunophenoscore predicts a better prognosis and better response to immunotherapy. In the present study, patients were grouped according to the expression of CTLA‐4 and PD‐1, in which the immunophenoscore of the high‐ and low‐risk patients were compared among subgroups to validate whether patients at different risks have different responses to ICIs treatment.

## 3 RESULTS

### 3.1 Identification of DEhypolncRNAs and construction of prognostic classifier

**FIGURE 1:**
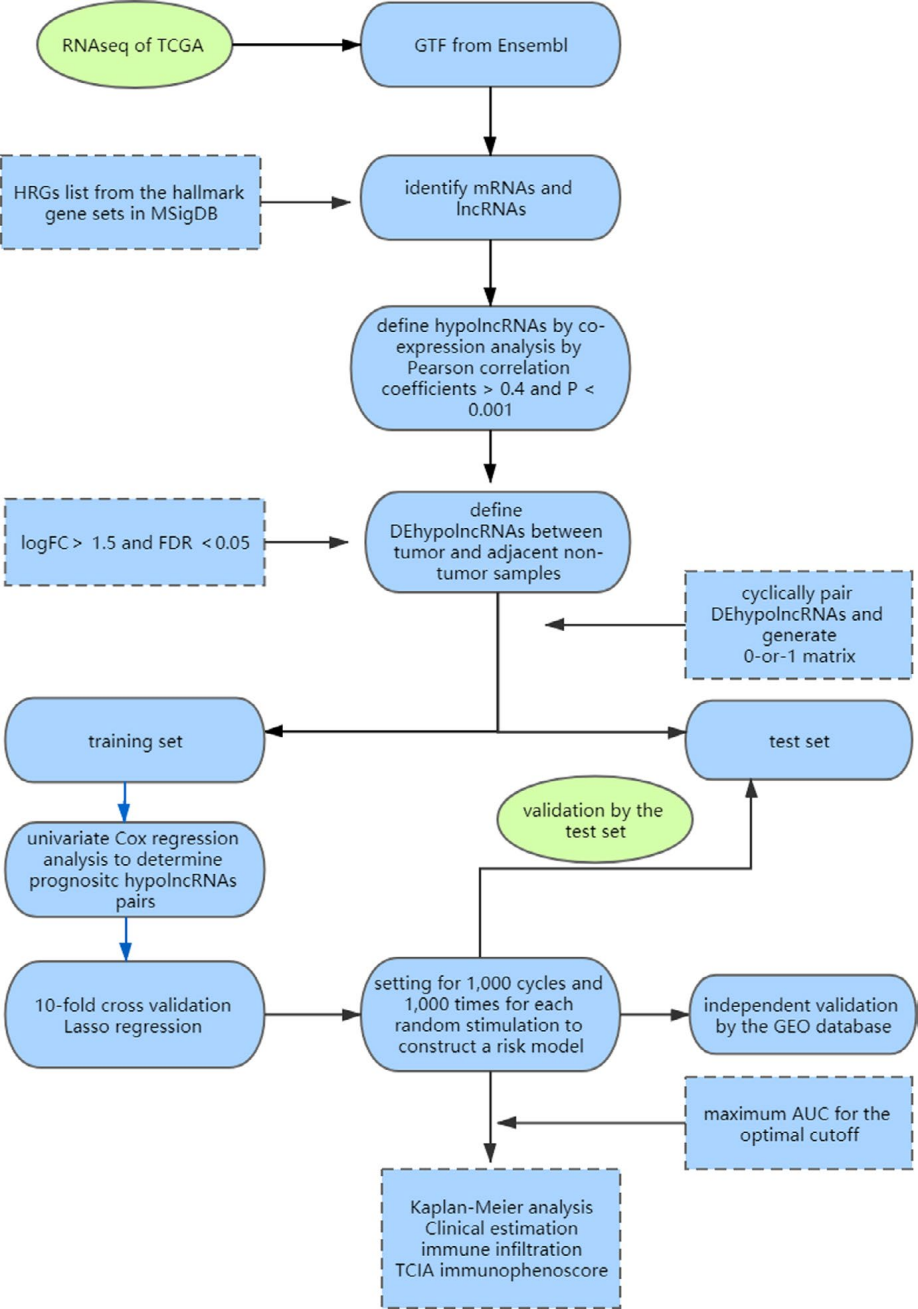
The workflow of the present study

**FIGURE 2:**
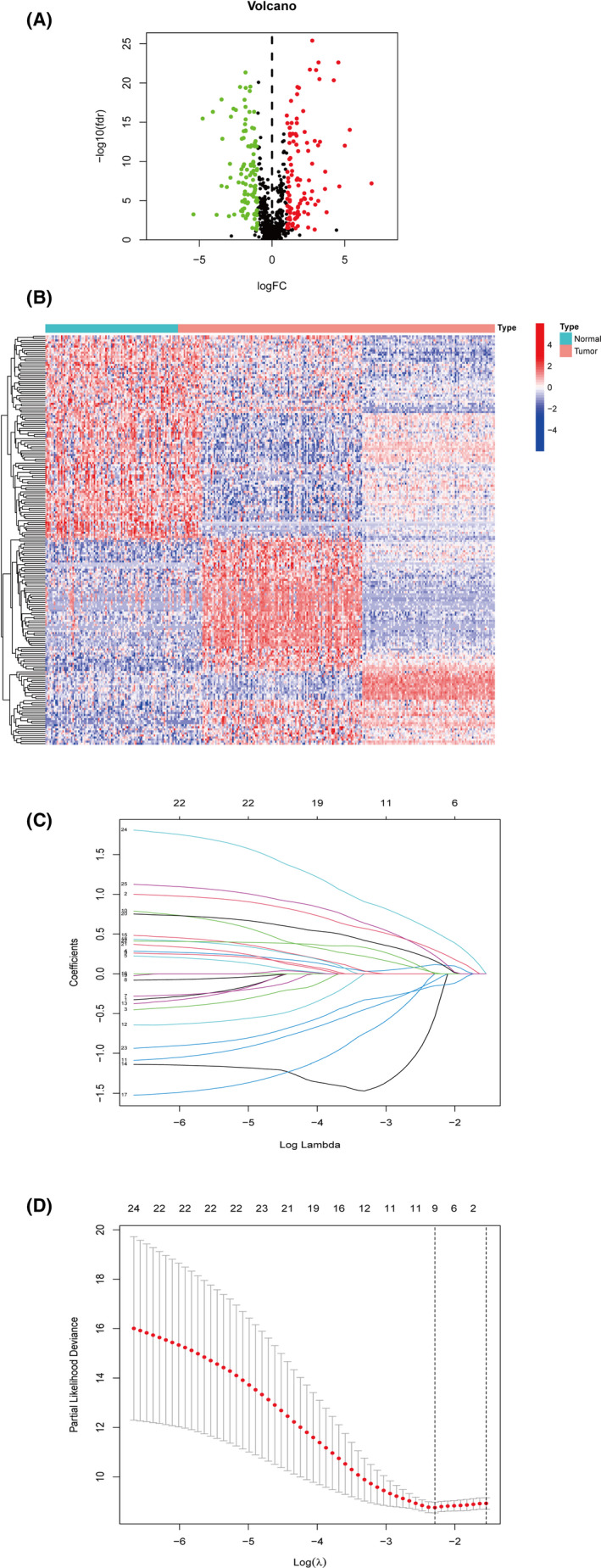
(A) The volcano plot of DEhypolncRNAs between tumor and adjacent non‐tumor samples. (B) The heatmap of DEhypolncRNAs between tumor and adjacent non‐tumor samples. (C) The Lasso regression was performed with the optimal value of λ. (D) Nine DEhypolncRNAs pairs were chosen by Lasso regression analysis

As shown in the workflow (Figure 1), 200 HRGs were extracted from MSigDB. A total of 1,4086 lncRNAs and 1,9604 mRNAs were identified, of which 864 hypolncRNAs were targeted depending on co‐expression analysis. A total of 106 downregulated and 103 upregulated DEhypolncRNAs were retrieved (Figure 2A and B). After cyclically pairing the DEirlncRNAs, a loop matrix was constructed and comprised of 1,7534 DEirlncRNAs pairs. The matrix was randomly divided into the train set and test set, and there was no statistical difference between the two groups in gender, age, and pathological stages (Table 1). The univariate Cox regression identified 25 prognostic DEhypolncRNAs pairs compressed to 9 DEhypolncRNAs pairs by Lasso regression analysis (Figure 2C and D) and multivariate Cox analysis and constructed the prognostic classifier. The formula of prognostic classifier was listed as follows: AL606489.1|AL512413.1 × 0.2986 + AL606489.1|AC024361.1 × 0.1159 + AL161431.1|LINC01010 × 0.0117 + AP004608.1|TUSC8 × −0.0847 + C2CD4D‐AS1|AC027288.3 × −0.5404 + AC104461.1|C20orf197 × 0.1638 + AC026471.3|HS1BP3‐IT1 × −0.1492 + AC245128.3|C20orf197 × 0.5289 + HS1BP3‐IT1|AC024361.1 × 0.2299.

**TABLE 1:** No statistical differences in terms of gender, age, and pathological stages in the train and test sets

Covariates	Type	Total	Train	Test	*p* value
Age	≤65	74 (43.02%)	30 (37.5%)	44 (47.83%)	0.2263
Age	>65	98 (56.98%)	50 (62.5%)	48 (52.17%)	
Gender	Female	60 (34.88%)	30 (37.5%)	30 (32.61%)	0.6094
Gender	Male	112 (65.12%)	50 (62.5%)	62 (67.39%)	
Stage	Stage I–II	129 (75%)	54 (67.5%)	75 (81.52%)	0.0522
Stage	Stage III–IV	43 (25%)	26 (32.5%)	17 (18.48%)	

**FIGURE 3:**
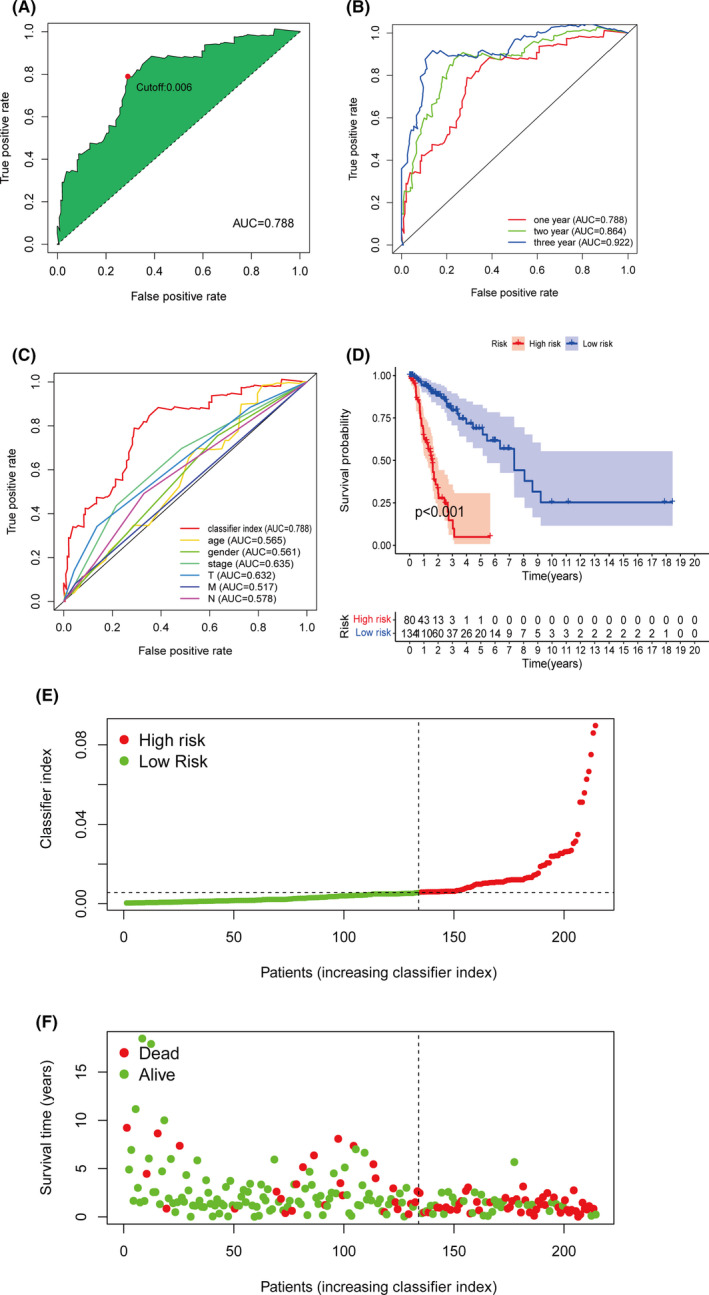
(A) The ROC curve achieved the maximum AUC when the cutoff point was set at 0.006, for which the optimal models were determined. (B) The ROC curve of the prognostic classifier in 1, 2, and 3 year. (C) The 1‐year ROC curves of diverse clinical parameters showed the predictive advantage of the prognostic classifier. (D) The Kaplan–Meier analysis of train set showed that the low‐risk patients have better prognosis than the high‐risk patients. (E and F) The classifier index and clinical outcome of the TCGA cohorts are shown

The AUCs of the ROC curve were shown in Figure 3A. The maximum AUCs referred to 0.788 when the cutoff value of 1‐year ROC referred to 0.006. Furthermore, 2‐year and 3‐year AUCs reached 0.864 and 0.922, which showed a high authenticity in predicting survival (Figure 3B). Meanwhile, 1‐year ROC curves of clinical parameters including age, gender, and pathological stage were also generated (Figure 3C). The AUCs of the classifier index were higher than other indicators, indicating a more accurate prognostic and predictive ability.

### 3.2 Clinical evaluation and validation of the prognostic classifier

**FIGURE 4:**
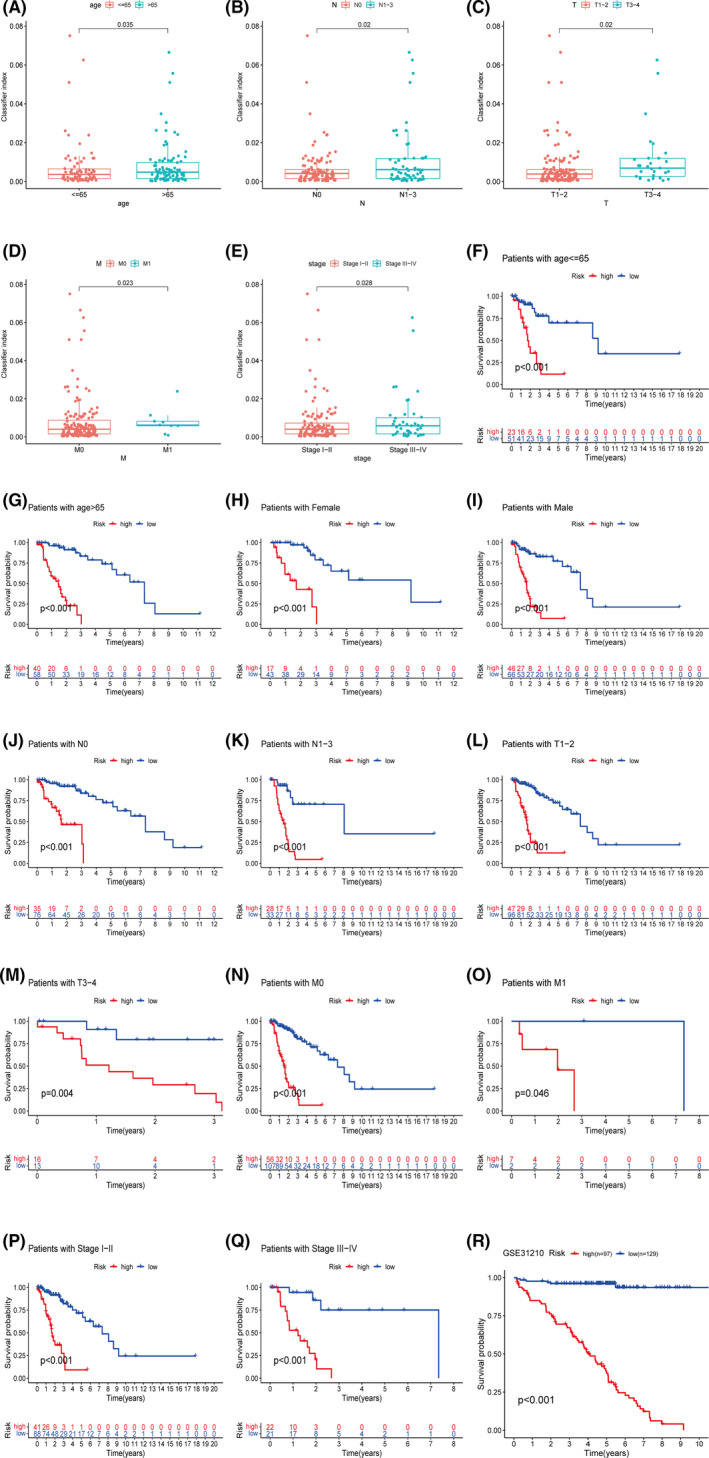
(A) Patients older than 65 years have higher classifier index than patients younger than 65 years. (B–E) The higher classifier index was shown in worse N stage, T stage, M stage, and pathological stages. (F–Q) The prognostic classifier well distinguished the high‐ and low‐risk patients for prognosis in diverse clinical parameters (age, gender, and pathological stages). (R) The survival analysis indicated that the prognostic classifier was validated in an independent cohort GSE31210

**FIGURE 5:**
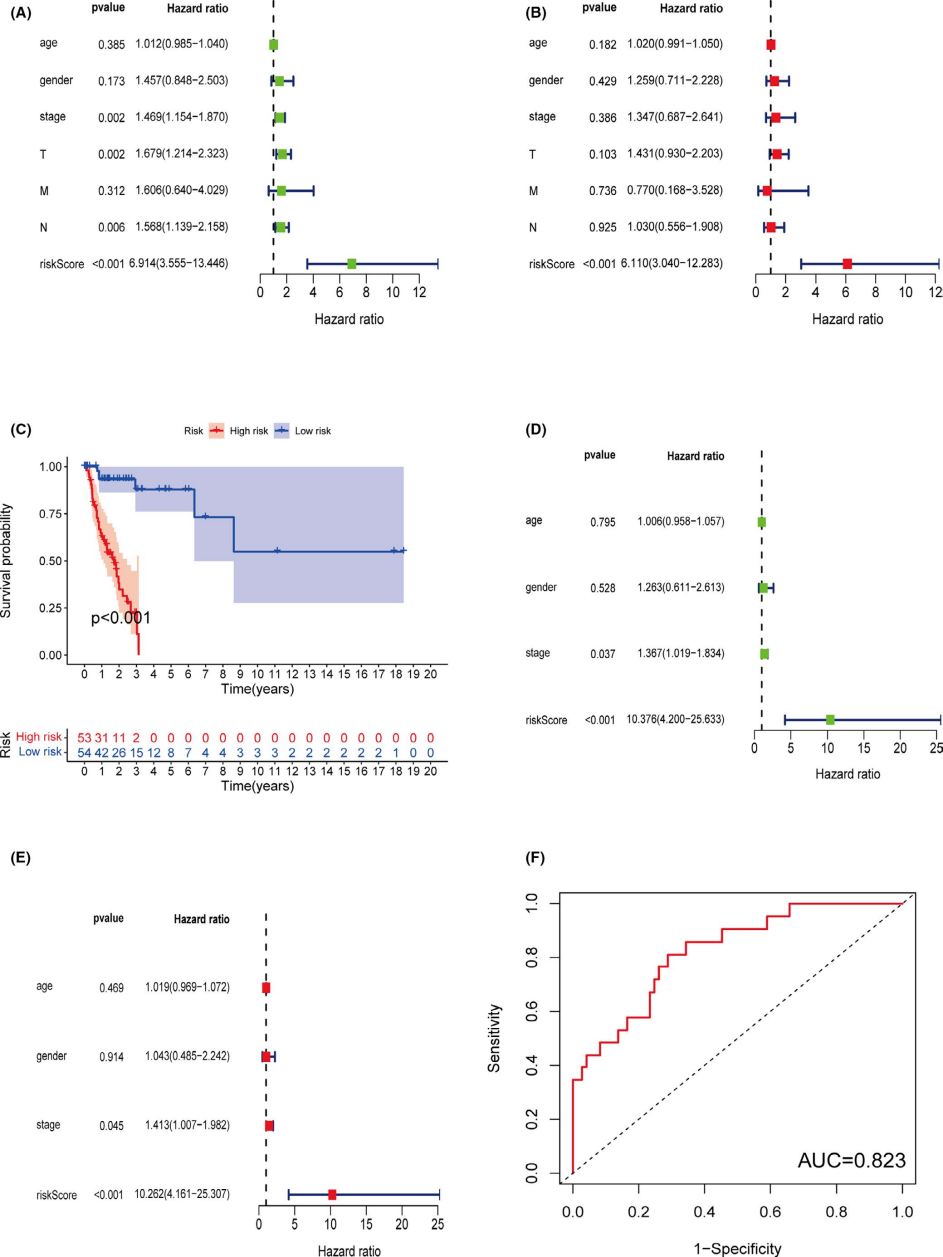
(A) The univariate Cox regression analysis showed the classifier was statistically significant for prognosis. (B) The multivariate Cox regression demonstrated the classifier presented as an independent prognostic predictor. (C) The survival analyses of the test set indicated that the prognostic classifier was validated in randomized grouping. (D and E) The univariate Cox regression and multivariate Cox regression analyses indicated the classifier served as an independent predictor for survival. (F) The AUC of the test set reached at 0.823

A total of 102 LUSC and LUAD patients were identified as low‐risk, and 112 patients were high‐risk, depending on the cutoff point (Figure 3E and F). The Kaplan–Meier analysis showed the prognosis of the low‐risk patients was superior to the high‐risk patients (Figure 3D). To further verify the validity of the prognostic classifier under different clinical conditions, we performed a *t*‐test and survival analysis under different clinical classifications. The classifier index was higher in older patients or patients with worse pathologic stages (Figure 4A–E). In young or old patients, female and male, and early or late stage, the prognostic classifier still clearly differentiated between different risk groups for prognosis (Figure 4F–Q). The univariate and multivariate Cox regression analyses demonstrated that the prognostic classifier was an independent predictor of prognosis (*p* < 0.001) (Figure 5A and B).

To further verify the accuracy of the prognostic classifier, patients in the test set were classified using the same formula. The low‐risk patients uniformly showed better outcomes (Figure 5C). Moreover, the classifier index was still an independent prognostic predictor in the test set (Figure 5D and E). The 1‐year AUCs reached 0.823 in the test set (Figure 5F).

**FIGURE 6:**
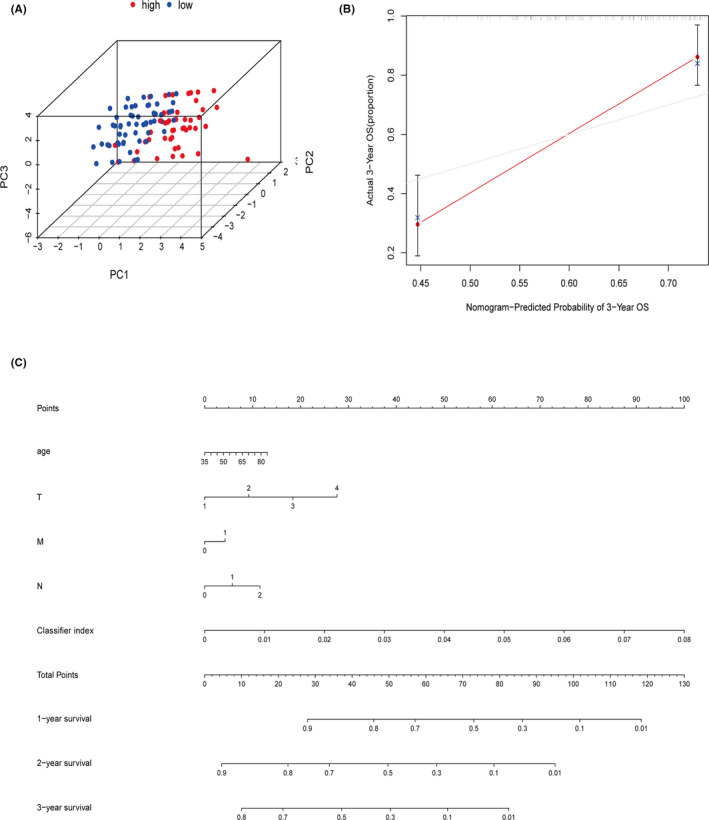
(A) The PCA showed the classifier could successfully distinguish double‐negative LUAD and LUSC patients at different risks. (B) The calibration plot of the nomogram indicating a good predictability of the classifier. (C) Nomogram of the classifier index and other clinical factors for predicting 1‐,2‐, and 3‐year survival in double‐negative LUAD and LUSC

The PCA plots revealed that the classifier index could clearly distinguish the high‐ and low‐risk patients (Figure 6A). Moreover, we used another quantitative method, nomogram, to predict the individual probability of survival, in which the classifier index was combined with clinical features. The nomogram calculated 1‐,2‐, and 3‐year survival rates for double‐negative LUAD and LUSC patients (Figure 6C). The calibration diagram showed that the predicted curve was in good agreement with the observed curve (Figure 6B), indicating the hypolncRNAs classifier had a great promise in predicting survival outcomes.

Further independent cohort verification was carried out in the Gene Expression Omnibus (GEO) database. GSE31210 contained RNA expression profiles and clinical data of 226 LUAD patients. Gene expression matrix including lncRNAs expression data was obtained after annotation by platform GPL570. A total of 97 patients were identified as the high‐risk and 129 as the low‐risk by the same formula. The survival analysis showed that the low‐risk patients had significantly better outcomes than the high‐risk patients (Figure 4R).

### 3.3 Functional enrichment analysis of DEHRGs

**FIGURE 7:**
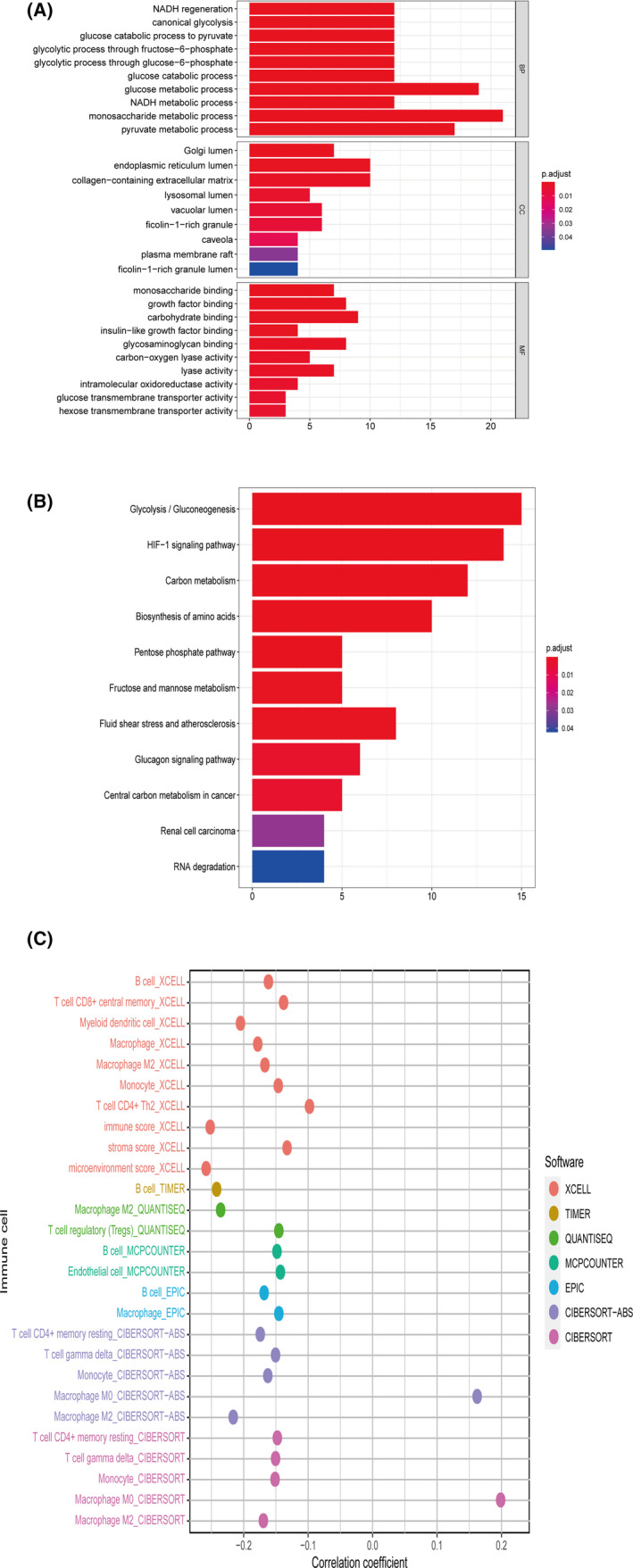
(A) The GO enrichment of DEHRGs. The count shows the number of enriched gene in specific pathways. The smaller the *p* value, the greater the authenticity of enrichment. Gene ratio refers to the ratio of the enriched HRGs number to the total number of DEHRGs. (B) KEGG enrichment of DEHRGs. The count shows the number of enriched gene in specific pathways. The smaller the *p* value, the greater the authenticity of enrichment. Gene ratio refers to the ratio of the enriched HRGs number to the total number of DEHRGs. (C) The classifier index was negatively associated with multiple tumor‐infiltrating immune cells shown by Spearman correlation analysis

Functional enrichment analysis of DEHRGs offered a biological understanding of related processes in double‐negative LUAD and LUSC. The GO terms (Figure 7A) for biological processes (BP) are enriched mainly in glucose metabolic processes, such as canonical glycolysis, glucose catabolic process to pyruvate, and glycolytic process through fructose−6−phosphate. The cellular components (CC) included golgi lumen, endoplasmic reticulum lumen, and collagen‐containing extracellular matrix. DEHRGs are mainly enriched in monosaccharide binding, growth factor binding, and carbohydrate binding with molecular function (MF). As for KEGG pathway enrichment analysis (Figure 7B), DEHRGs were significantly related to glycolysis/gluconeogenesis, HIF‐1 signaling pathway, and carbon metabolism.

### 3.4 Integrated analysis for tumor‐infiltrating immune cells revealed immunosuppressive status with the prognostic classifier

Based on current acknowledged methods, immune infiltration fluctuations were revealed between groups. The negative correlation coefficients were widely observed, which meant an immunosuppression state in higher classifier index patients. Multiple immune cell types of XCELL, including B cell, T‐cell CD8+ central memory, myeloid dendritic cell, macrophage, macrophage M2, monocyte, and T‐cell CD4+ Th2 were negatively correlated with the classifier index. T‐cell CD4+ memory resting, T‐cell gamma delta, monocyte, and macrophage M2 in CIBERSORT‐ABS, T‐cell CD4+ memory resting, T‐cell gamma delta, monocyte, and macrophage M2 in CIBERSORT, B cell and macrophage in EPIC, macrophage M2 and T‐cell regulatory (Tregs) in QUANTISEQ, B cell and endothelial cell in MCPCOUNTER, and B cell in TIMER also showed negative correlations with classifier index (Figure 7C).

**FIGURE 8:**
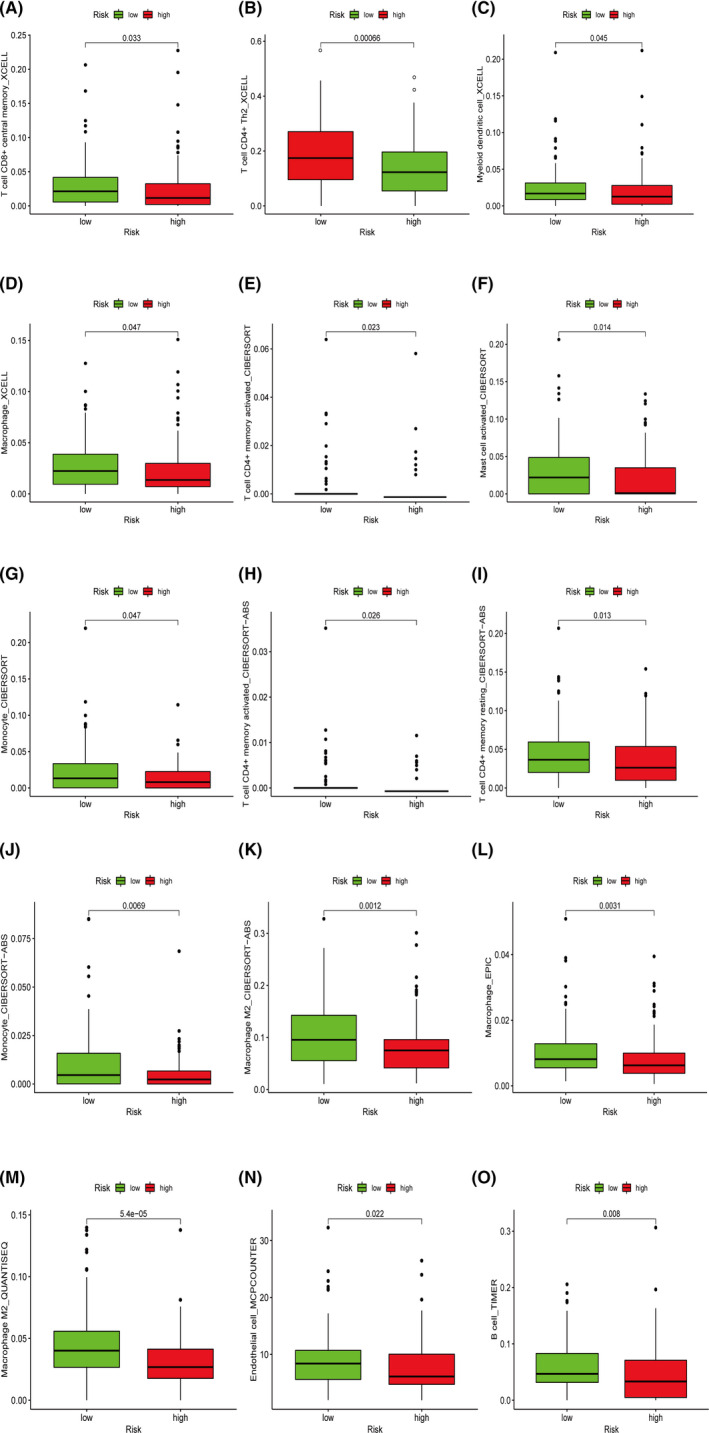
The Wilcoxon signed‐rank test revealed lower levels of T‐cell CD8+ central memory, T‐cell CD4+ Th2, myeloid dendritic cell, and macrophage in XCELL (A–D), T‐cell CD4+ memory activated, mast cell activated, and monocyte in CIBERSORT (E–G), T‐cell CD4+ memory activated, T‐cell CD4+ memory resting, monocyte, and macrophage M2 in CIBERSORT‐ABS (H–K), macrophage in EPIC (L), macrophage M2 in QUANTISEQ (M), endothelial cell in MCPCOUNTER (N), and B cell in TIMER (O), indicating an immunosuppression status in the high‐risk patients

The Wilcoxon signed‐rank test obtained similar results. T‐cell CD8+ central memory, T‐cell CD4+ Th2, myeloid dendritic cell, and macrophage in XCELL (Figure 8A–D), T‐cell CD4+ memory activated, mast cell activated, and monocyte in CIBERSORT (Figure 8E–G), T‐cell CD4+ memory activated, T‐cell CD4+ memory resting, monocyte, and macrophage M2 in CIBERSORT‐ABS (Figure 8H–K), macrophage in EPIC (Figure 8L), macrophage M2 in QUANTISEQ (Figure 8M), endothelial cell in MCPCOUNTER (Figure 8N), and B cell in TIMER (Figure 8O) showed lower level in the high‐risk patients.

**FIGURE 9:**
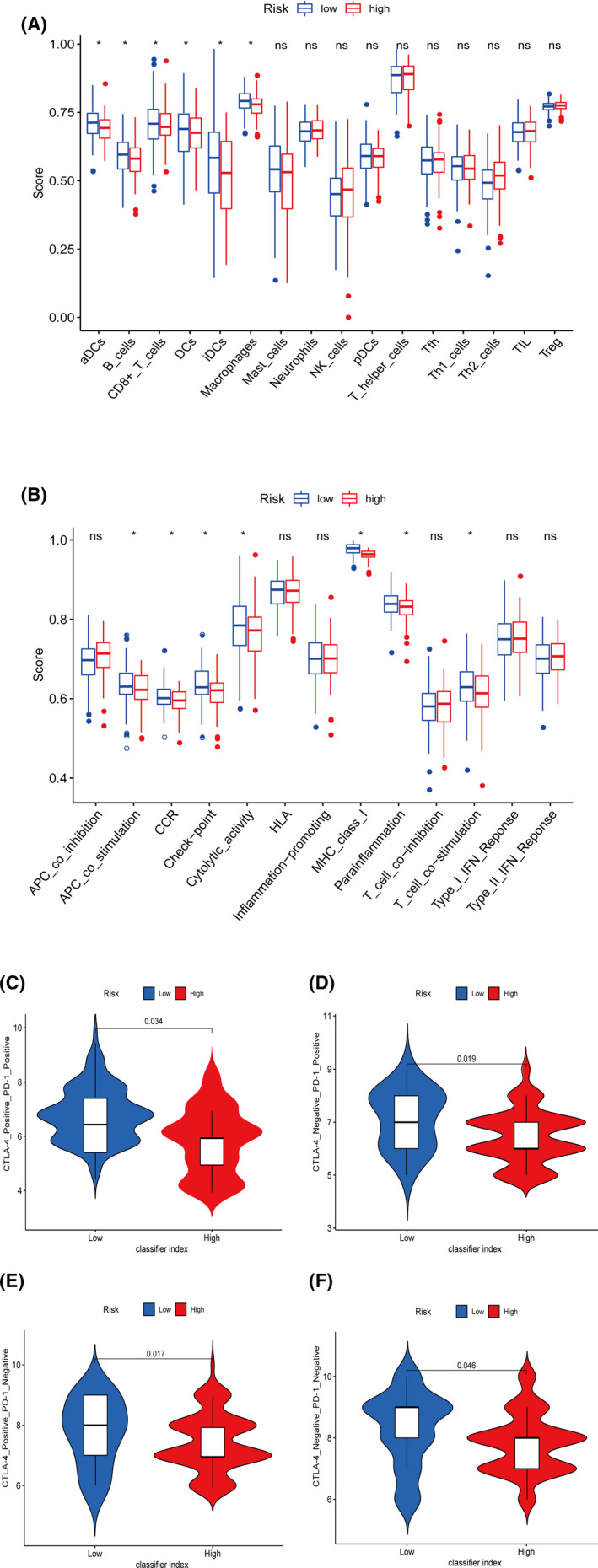
(A) The immune‐related functional cells between the high‐ and low‐risk patients. (B) The immune pathway functions between the high‐ and low‐risk patients. (C–F) The low‐risk patients showed lower classifier indexes in all four subgroups as CTLA‐4 positive PD‐1 positive, CTLA‐4 negative PD‐1 positive, CTLA‐4 positive PD‐1 negative, and CTLA‐4 negative PD‐1 negative

Previously, we found the immunosuppression state and survival disadvantage with the high‐risk patients, consistent with the immune functions and pathways analysis in ssGSEA. Among the 29 immune gene sets, immune‐related functional cells such as aDCs, aDCs, B cells, CD8+ T cells, DCs, iDCs, and macrophages showed lower ssGSEA scores in the high‐risk patients (Figure 9A). Similarly, immune pathways such as APC co‐stimulation, CCR, Check‐point, cytolytic activity, MHC class I, para‐inflammation, and T‐cell co‐stimulation gained lower ssGSEA scores in the high‐risk group (Figure 9B).

### 3.5 Assessment of the immunophenoscore from TCIA to predict the response to ICIs

In the previous steps, we proved the negative correlation between the classifier index and the immune‐infiltrating cells, which directly indicated the immunosuppression status of the high‐risk patients. We further determined the relationship between the classifier index and immunotherapy. TCIA is a web‐based database (https://tcia.at/home) that provides information on the cellular composition of tumor‐infiltrating lymphocytes (TILs) and the response to checkpoint‐blocking immunotherapies for 20 solid cancers in TCGA. TCIA generates a comprehensive score, the immunophenoscore, to reveal the tumor immune heterogeneity of 20 valid cancers as an indication of the tumor cell types that may be susceptible to ICIs treatment. Higher immunophenoscore was positively associated with better anti‐CTLA‐4 and anti‐PD‐1 treatment responses. According to the expression of CTLA‐4 and PD‐1, patients were divided into four groups as CTLA‐4 positive PD‐1 positive, CTLA‐4 negative PD‐1 positive, CTLA‐4 positive PD‐1 negative, and CTLA‐4 negative PD‐1 negative. The high‐ and low‐risk patients showed a distinct difference of immunophenoscore for all four subgroups. The low‐risk patients all had higher immunophenoscores, suggesting that the prognostic could predict responses to immunotherapy regardless of CTLA‐4 and PD‐1 expressions (Figure 9C–F).

## 4 DISCUSSION

Previous studies have attempted to construct lncRNAs predictors to assess cancer risk, mostly based on quantifying the transcripts level directly.^23^, ^24^ However, the difference of detection method and platform will lead to the fluctuations of expression values. We generated a particular approach to construct the 0 or 1 matrix by an interaction loop to address the batch effect's bias. The raw profiles of RNA sequencing were retrieved from TCGA, of which the co‐expression relationship between lncRNAs and mRNAs was determined. By cyclically pairing DEhypolncRNAs, the interaction loop of hypolncRNA pairs was accomplished, and candidate prognostic hypolncRNA pairs were screened out to build the prognostic classifier. The optimal cutoff value was chosen by counting each point on the AUC of the ROC curve to divide the high‐ and low‐risk patients. In addition to validation of the prognostic classifier in the test set, we also selected an independent cohort for validation in the GEO database. We further evaluated the classifier under various conditions with other clinical parameters such as age, gender, and pathological stages. Our classifier was able to identify the high‐risk patients among different ages, genders, and pathological stages.

lncRNAs are very versatile molecules that can drive many cancer‐related phenotypes directly or indirectly to promote or inhibit the expression of protein‐coding genes. Recent studies indicate that lncRNAs are also deeply involved in developing and activating immune cells, especially in the tumor immune microenvironment.^25^, ^26^ Tumor‐infiltrating immune cell pathways such as the differentiation and exhaustion of T cells or the immunodeficiency of natural killer cells were affected by lncRNAs.^17^, ^24^ Hypoxia is common in malignancy and can promote the invasive behavior of tumors.^27^ Metabolic adaptations of tumor cells to hypoxia, such as increased glucose uptake and lactate production, also promote and maintain an immunosuppressive tumor microenvironment.^13^, ^28^ The immunosuppression may be induced by inhibiting T cells or promote T‐cell death.^29^ HRGs such as hypoxic‐inducible factor 1α (HIF1A) can promote the expression of PD‐L1 in mouse models of cancer.^30^ Moreover, multiple HRGs (such as HIF1A, VEGFA, GLUT1, and CAIX) were correlated with PD‐L1 expression in LUAD.^31^ The combination of HRGs and lncRNAs can more effectively reveal the immune characteristics of double‐negative LUAD and LUSC. Proper combination strategies of HRGs and lncRNAs can even predict responses to immunotherapy, which have been proved in immunophenoscore analysis based on TCIA. Common acceptable methods including TIMER,^32^, ^33^ CIBERSORT,^34^, ^35^ XCELL,^36^, ^37^ QUANTISEQ,^38^, ^39^ MCPcounter,^40^ EPIC,^41^ and CIBERSORT‐ABS^42^ were used to analyze the relationship between classifier index and tumor‐infiltrating immune cells, which were thought to impact the treatment response of ICIs. For example, a higher CD8+ T‐cell infiltration predicts a better response from pembrolizumab.^43^ Tumor‐infiltrating T lymphocytes were positively associated with PD‐L1 expression and survival time in various tumor types.^44^, ^45^, ^46^ CD4+ memory T cells and B cells are localized and enriched in tertiary lymphoid structures, which have shown benefits on many tumor types.^47^, ^48^ Memory B cells in both naïve and memory T‐cell response as antigen‐presenting cells, thus inducing an antitumor immune response.^49^ In our analysis, multiple types of immune cells, including CD8+ T cells and CD4+ T cells, were negatively related to the classifier index and down‐expressed in the high‐risk patients, explaining the potential remodeling of tumor immune environment in double‐negative LUAD and LUSC. We assumed an immunosuppression state in the high‐risk patients, and the classifier could be used as a predictor of immunotherapy benefits.

Some of the lncRNAs involved in the classifier have been studied in previous research. TUSC8 and LINC01010 are reported to act as a suppressor in the invasion of multiple cancer cells in different pathways.^50^, ^51^, ^52^, ^53^ Multiple signatures that predict tumor prognosis also involve LINC01010.^53^, ^54^, ^55^ AL161431.1 facilitates tumor cell proliferation and migration in endometrial carcinoma.^56^ lncRNAs signature containing AL161431.1 can serve as a prognosis predictor in LUSC.^57^ However, most lncRNAs involved in our classifier have not been reported in other lung cancer studies. This suggested that the current studies focus on double‐negative LUAD and LUSC have not paid enough attention to lncRNAs, especially hypolncRNAs, hinting at some critical mechanisms that have been overlooked.

## 5 CONCLUSION

In general, we proposed a prognostic classifier based on HRGs and lncRNAs. Not only can the high‐risk patients be distinguished by the classifier under various clinical conditions, but also different tumor immune microenvironments can be identified. This finding may serve as a potential guide to targeted immunotherapy and provide ideas for the further development of new immunotherapies. Given the nature of our primary data, we would recommend multi‐center verification for the prognostic value of the hypolncRNAs classifier and potential use in EGFR inhibitors or anti‐PD‐1/PD‐L1 treatment.

## CONFLICT OF INTEREST

All the authors declared no competing interest.

## AUTHOR CONTRIBUTIONS

Fang Zhao and Jie Zhu designed the study and wrote the manuscript. Min Wang acquired and analyzed the data and reviewed the manuscript. All the authors participated in discussion of related data and approved the final manuscript. Fang Zhao and Min Wang are the co‐first authors of this article.

## ETHICAL STATEMENT

The TCGA database and GEO database are publicly available, and the present study was performed based on the guideline of these databases. All patient information was anonymized and de‐identified in the TCGA database. Thus, we were exempted from the ethics committee approval and patients’ informed consent.

## Data Availability

Publicly available datasets were analyzed in this study. These data can be found here: https://tcga‐data.nci.nih.gov/tcga/ and https://www.ncbi.nlm.nih.gov/geo/. They can also be obtained from the corresponding author by reasonable request.
